# Sc-ncDNAPred: A Sequence-Based Predictor for Identifying Non-coding DNA in *Saccharomyces cerevisiae*

**DOI:** 10.3389/fmicb.2018.02174

**Published:** 2018-09-12

**Authors:** Wenying He, Ying Ju, Xiangxiang Zeng, Xiangrong Liu, Quan Zou

**Affiliations:** ^1^School of Computer Science and Technology, Tianjin University, Tianjin, China; ^2^School of Information Science and Technology, Xiamen University, Xiamen, China; ^3^Shandong Provincial Key Laboratory of Biophysics, Institute of Biophysics, Dezhou University, Dezhou, China

**Keywords:** non-coding DNA, DNA sequence, feature representation, genome synthesis, support vector machine

## Abstract

With the rapid development of high-speed sequencing technologies and the implementation of many whole genome sequencing project, research in the genomics is advancing from genome sequencing to genome synthesis. Synthetic biology technologies such as DNA-based molecular assemblies, genome editing technology, directional evolution technology and DNA storage technology, and other cutting-edge technologies emerge in succession. Especially the rapid growth and development of DNA assembly technology may greatly push forward the success of artificial life. Meanwhile, DNA assembly technology needs a large number of target sequences of known information as data support. Non-coding DNA (ncDNA) sequences occupy most of the organism genomes, thus accurate recognizing of them is necessary. Although experimental methods have been proposed to detect ncDNA sequences, they are expensive for performing genome wide detections. Thus, it is necessary to develop machine-learning methods for predicting non-coding DNA sequences. In this study, we collected the ncDNA benchmark dataset of *Saccharomyces cerevisiae* and reported a support vector machine-based predictor, called Sc-ncDNAPred, for predicting ncDNA sequences. The optimal feature extraction strategy was selected from a group included mononucleotide, dimer, trimer, tetramer, pentamer, and hexamer, using support vector machine learning method. Sc-ncDNAPred achieved an overall accuracy of 0.98. For the convenience of users, an online web-server has been built at: http://server.malab.cn/Sc_ncDNAPred/index.jsp.

## Introduction

After the implementation of many whole genome sequencing projects, more and more researches showed that non-coding DNA (ncDNA) is a major component of the biological genome. Numerous studies (Vogel, 1964; Thomas, 1971; Eddy, 2012; Puente et al., 2015; Liu et al., 2017a; Yao et al., 2018) have shown that the complexity of organisms is related to the length of non-coding regions, which are specially transcribed in physiological and disease states. Although the function of most ncDNAs is still unknown(Khurana et al., 2016), some studies (Horn et al., 2013; Huang et al., 2013; Vinagre et al., 2013; Puente et al., 2015; Hu et al., 2017, 2018; Rheinbay et al., 2017; Liao et al., 2018; Zhang W. et al., 2018) have shown that most cancer-related gene mutations are located in ncDNA regions. How ncDNAs specifically affect tumor formation is also an urgent problem to be solved. In addition, ncDNAs in the genome play an important role in gene expressing, regulatory, and inheritance (Khurana et al., 2016).

Especially, with the rapid growth and development of synthetic biology, research in the genomics is advancing from genome sequencing to genome synthesis (Erlich and Zielinski, 2017; Jain et al., 2018; Liu B. et al., 2018). In recent years, various DNA assembly technologies (Ni et al., 2017; Wu et al., 2017; Xie et al., 2017; Zhang et al., 2017b) have been developed according to the principles of atypical enzyme cut connection (Engler et al., 2009; Sleight et al., 2010), single strand annealing and splicing (Gibson et al., 2009; Li and Elledge, 2012) and PCR (Warrens et al., 1997), which provide more rapid technical support for synthetic biology. In the following years, people are committed to improving the efficiency of large scale DNA assembly technologies. With the rapid development of the computer network and the popularity of the Internet, the number of digital information, such as network data, audio data, and video data, is increasing rapidly. It is urgent to establish a new system which has more efficiency than the existing storage system. DNA storage technology (Baum, 1995; Davis, 1996; Carr and Church, 2009) can meet the requirements above. In a new study (Shipman et al., 2017), the researchers introduced a method that encode images and video images into the genome of the *Escherichia coli* and read the corresponding images and videos from the genome of living bacterial cells. All the above studies require a large amount of DNA data.

As a complex type of genetic information, DNA sequences have specific characteristics not only in the coding sequence (cDNA) but also in the ncDNA sequences. Currently, the identification of cDNAs and ncDNAs relies mainly on experimental methods. However, traditional experimental methods are time-consuming and laborious, and the amount of genomic data is large and the sequence types are complex. In this context, there is an urgent need to establish accurate and efficient prediction methods to mine the information and knowledge of ncDNAs and cDNAs. Computational methods, which achieve a complementary effect, indeed effectively improved the recognition accuracy (Zhou et al., 2016).

**Figure 1:**
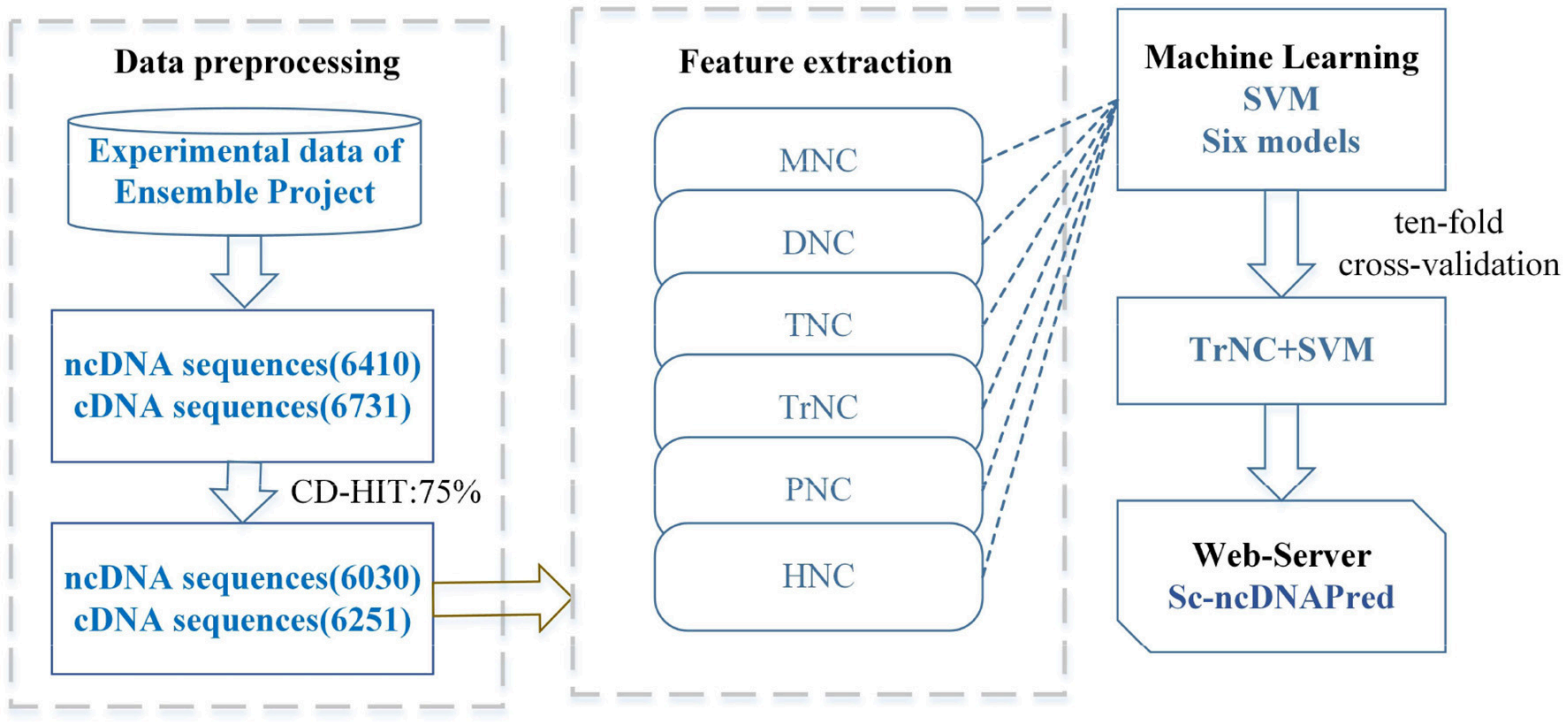
The workflow of Sc-ncDNAPred.

In this study, a SVM-based computational method was first established to recognize the ncDNA sequences in *Saccharomyces cerevisiae (S. cerevisiae)*. Totally several types of features, such as mononucleotide composition (MNC), dimer nucleotide composition (DNC), trimer nucleotide composition (TNC), tetramer nucleotide composition (TrNC), pentamer nucleotide composition (PNC), and hexamer nucleotide composition (HNC) were extracted. The optimal feature extraction strategy was selected using SVM machine learning method. The workflow of constructing the Sc-ncDNAPred model is shown in Figure 1.

## Methods

### Benchmark dataset

In this study, the benchmark dataset was derived from the Ensembl genome database project (Hubbard et al., 2002), which is one of several well-known genome browsers for the retrieval of genomic information. Experimentally validated cDNA sequences of *S. cerevisiae* were extracted from their database, which contains 6713 samples. Intercepting the ncDNAs of the *S. cerevisiae* based on the initial marker information of the coding region provided by the original genomic data. By doing so, we obtained 6410 ncDNA samples. To get rid of redundancy, the CD-HIT (Li and Godzik, 2006) was adopted to remove those sequences that had ≥ 75% sequence identity. Finally, we obtained 6030 and 6251 samples in ncDNAs and cDNAs, respectively. Thus, the benchmark dataset can be formulated as

(1) $$\begin{array}{c} S=S^{+}\cup^{\text{​}}S^{-} \end{array}$$

where *S*^+^ contained 6030 ncDNA samples, *S*^−^contained 6251 cDNA samples and the symbol ∪ means the ‘union' in the set theory.

**Figure 2:**
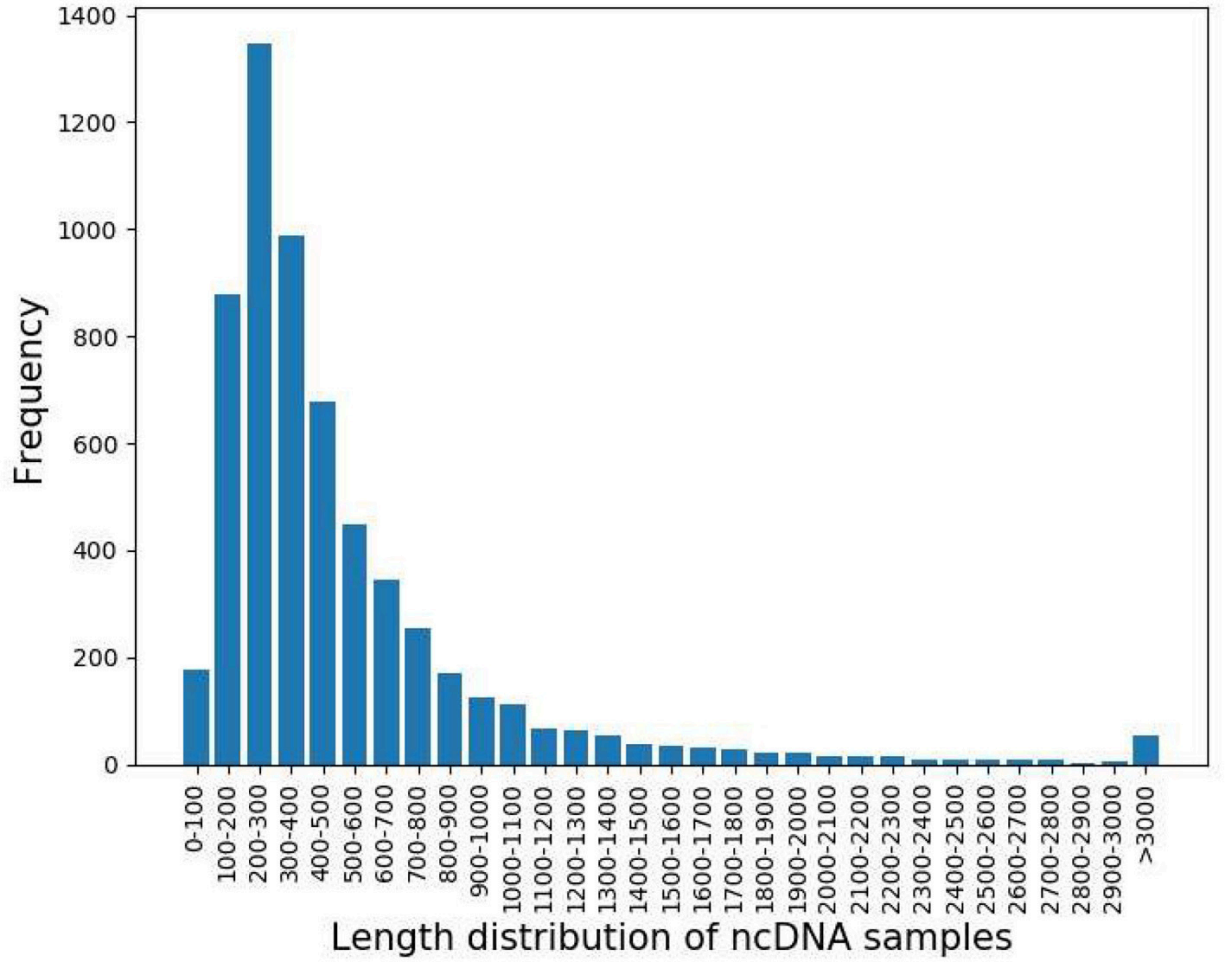
The length distribution of ncDNA samples.

The length distribution of ncDNA samples was shown in Figure 2. According to the graph, the length distribution of ncDNA is mainly between 100 and 800.

### Feature vector construction

A sample can be simplified by a convenience form as:

(2) $$\begin{array}{c} \text{P}={\text{R}}_{1}{\text{R}}_{2}{\text{R}}_{3}{\text{R}}_{4}\;\ldots {\text{ R}}_{L-1}{\text{R}}_{L} \end{array}$$

where R_*i*_ (*i* = 1,2,3 … *L*) represents the nucleotide at *i*-th position in one sequence.

### K-mer composition

*K*-mer nucleotide composition has been applied in many fields of bioinformatics (Liu et al., 2015b,c; Kim et al., 2017; Matias Rodrigues et al., 2017; Orenstein et al., 2017; Liu, 2018; Liu X. et al., 2018; Rangavittal et al., 2018). MNC equate to *k* = 1, DNC equate to *k* = 2, TNC equate to *k* = 3, TrNC equate to *k* = 4, PNC equate to *k* = 5, HNC equate to *k* = 6. The occurrence frequency of *k*−*mer*(*i*)can be represented as:

(3) $$\begin{array}{l} \begin{array}{ccc} f_{i}^{k} & = & f(k-mer(i))=\frac{n_{i}^{k}}{L-k+1} \end{array}\\ \;\begin{array}{c} \left(i=1,2,\ldots ,4^{k};k=1,2,3,4,5,6\right) \end{array} \end{array}$$

where $n_{i}^{k}$ denote the number of the *i*-th *k*-mer, *L* is the length of the sample sequence. Thus, each DNA sample can be defined feature vectors in different dimension of size 4^*k*^. The generalized form of whole feature vectors *X* can be given by:

(4) $$X={\left[f_{1}^{k},f_{2}^{k},\cdots \;,f_{i}^{k},\cdots f_{4^{k}}^{k}\right]}^{T}$$

### Feature ranking

Each sample sequence was represented by a large set of features, which leads to the redundant information (Wei and Billings, 2007; Senawi et al., 2017). In order to distinguish the contribution of different features to the prediction model. To analyze these feature vectors, *F-score* method (Chen W. et al., 2016; Jia and He, 2016; Tang et al., 2016, 2018; He and Jia, 2017) was adopted to rank the feature, in this study. The *F-score* value of the *i*-th feature is defined as:

(5) $$F-score(i)=\frac{{\left({\bar{x}}_{i}{}^{\left(+\right)}-{\bar{x}}_{i}\right)}^{2}\text{ + }{\bar{x}}_{i}{}^{\left(-\right)}-{\bar{x}}_{i}{}^{2}}{\frac{1}{n^{+}-1}\sum_{k=1}^{n^{+}}{\left(x_{k,i}^{\left(+\right)}-{\bar{x}}_{i}{}^{\left(+\right)}\right)}^{2}+\frac{1}{n^{-}-1}\sum_{k=1}^{n^{-}}{\left(x_{k,i}^{\left(-\right)}-{\bar{x}}_{i}{}^{\left(-\right)}\right)}^{2}}$$

where ${\overset{-}{x}}_{i}$, ${{\overset{-}{x}}_{i}}^{\left(+\right)}$ and ${{\overset{-}{x}}_{i}}^{\left(-\right)}$ are the average values of the *i*-th feature in whole, ncDNA and cDNA datasets, respectively. *n*^+^represents the number of ncDNA training samples, *n*^−^represents the number of cDNA training samples, $x_{k,i}^{\left(+\right)}$represents the *i*-th feature of the *k*-th ncDNA sample and$x_{k,i}^{\left(-\right)}$ represents the *i*-th feature of the *k*-th cDNA sample. Obviously, the feature with a greater score value indicates that it has a better discrimination ability.

### Support vector machine

Support vector machine (SVM) (Hearst et al., 1998) is a widely used two-class classification algorithm based on statistical learning theory. It has been proven to be powerful in many fields of pattern recognition and data classification (Byun and Lee, 2002; Nasrabadi, 2007; Zhang N. et al., 2018;). More and more applications also proved that SVM also has strong data processing capabilities in the fields of bioinformatics (Xiong et al., 2011; Jia et al., 2013, 2017; Cao et al., 2014; Liu et al., 2014, 2017b; Wei et al., 2015; Chen X. X. et al., 2016; Jia and He, 2016; Yang et al., 2016; Zou et al., 2016; Xiao et al., 2017; Qiao et al., 2018; Su et al., 2018). A set of ncDNA samples and cDNA samples were represented by the feature vectors. The SVM classifies the data by mapping the input feature vectors to a high-dimensional feature space using a kernel function. In this study, the public LIBSVM package (Chang and Lin, 2011) was implemented to train models for discriminating between ncDNA sequences and cDNA sequences. Here, the radial basis function (RBF) $K\left(S_{i},S_{j}\right)=exp\left(-\gamma {\left||S_{i}-S_{j}|\right|}^{2}\right)$ was set as the kernel function. The penalty parameter *C* and kernel parameter were preliminarily optimized through a grid search strategy.

### Performance evaluation

K-fold cross-validation (Chou and Zhang, 1995; Kohavi, 1995; Zhang et al., 2012a,b, 2015; Liu et al., 2015a; Chen X. et al., 2016; Li et al., 2016; Luo et al., 2016; Chen et al., 2017b, 2018a,b; Pan et al., 2017a; Xu et al., 2017; He et al., 2018) is one of the widely used approach to examine the ability of prediction model, and other approaches: independent dataset test and jackknife test (Chou and Shen, 2008) are also used in many applications. To reduce the computational cost, 10-fold cross validation was used to examine each model for its effectiveness in identifying ncDNA sequences. The training dataset were randomly divided into 10 subsets of approximately the same size. In each iteration, one subset was chosen as the test set and the remaining 9 subsets were used to train the model. For a complete cycle of a 10-fold cross-validation, the process was repeated 10 times until each subset was chosen as a test set. This 10-fold cross-validation procedure was repeated five times, then the results were averaged.

To evaluate the prediction performance of the models, five classic metrics were computed (Chou, 2001; Qiu et al., 2015, 2016; Liu et al., 2017; Pan et al., 2017b; Zhang et al., 2017a; Tang et al., 2018; Yang et al., 2018), including sensitivity (Sn), specificity (Sp), accuracy (Acc), Matthew correlation coefficient (MCC), and the receiver operating characteristic (ROC). These measurements were defined as:

(6) $$\begin{array}{l} \begin{array}{c} \begin{array}{ccc} Sn & = & 1-\frac{N_{-}^{+}}{N^{+}}\\ Sp & = & 1-\frac{N_{+}^{-}}{N^{-}}\\ Acc & = & \begin{array}{l} 1-\frac{N_{-}^{+}+N_{+}^{-}}{N^{+}+N^{-}}\;\\ \; \end{array} \end{array} \end{array}\\ \begin{array}{ccc} MCC & = & \frac{1-(\frac{N_{-}^{+}}{N^{+}}+\frac{N_{+}^{-}}{N^{-}})}{\sqrt{\left(1+\frac{N_{+}^{-}-N_{-}^{+}}{N^{+}})(1+\frac{N_{-}^{+}-N_{+}^{-}}{N^{-}}\right)}} \end{array} \end{array}$$

In these expressions, *N*^+^ and *N*^−^ are the total number of ncDNA and cDNA samples, respectively, while $N_{-}^{+}$ and $N_{+}^{-}$ are respectively the number of ncDNA samples incorrectly predicted as cDNA samples, and the number of cDNA samples incorrectly predicted as ncDNA samples.

## Results and discussion

### Prediction results of models

We used six types of effective feature extraction methods, such as MNC, DNA, TNC, TrNC, PNC, and HNC, as input of SVM to establish six models. The ability of each feature extraction method to discriminate between ncDNA and cDNA samples was compared by the 10-fold cross-validation (Table 1). As we can see from Table 1, the model for a combination SVM and TrNC yielded the best prediction performance, with the accuracy of 98.26%, the sensitivity of 98.01%, the specificity of 98.51%, and the MCC of 0.965, respectively. Then, the following second best prediction performance was yielded by TNC with the accuracy of 96.93%, the sensitivity of 96.62%, the specificity of 97.22%, and the MCC of 0.939, respectively. Besides, in the case of PNC, the corresponding model still obtained a good prediction results, which are 95.56% of accuracy, 95.25% of sensitivity, 95.84% of specificity and 0.911 of MCC, respectively.

**Table 1:** The 10-fold cross-validation results by different feature methods on the benchmark dataset.

**Methods**	**Sn (%)**	**Sp (%)**	**ACC (%)**	**MCC**
MNC	80.56	87.02	83.85	0.678
DNC	92.64	92.62	92.64	0.853
TNC	96.62	97.22	96.93	0.939
TrNC	98.01	98.51	98.26	0.965
PNC	95.25	95.84	95.56	0.911
HNC	90.71	92.25	91.49	0.830
All Features	95.99	96.08	96.03	0.921

*The experiments have been executed 5 times and the results were the mean values*.

**Figure 3:**
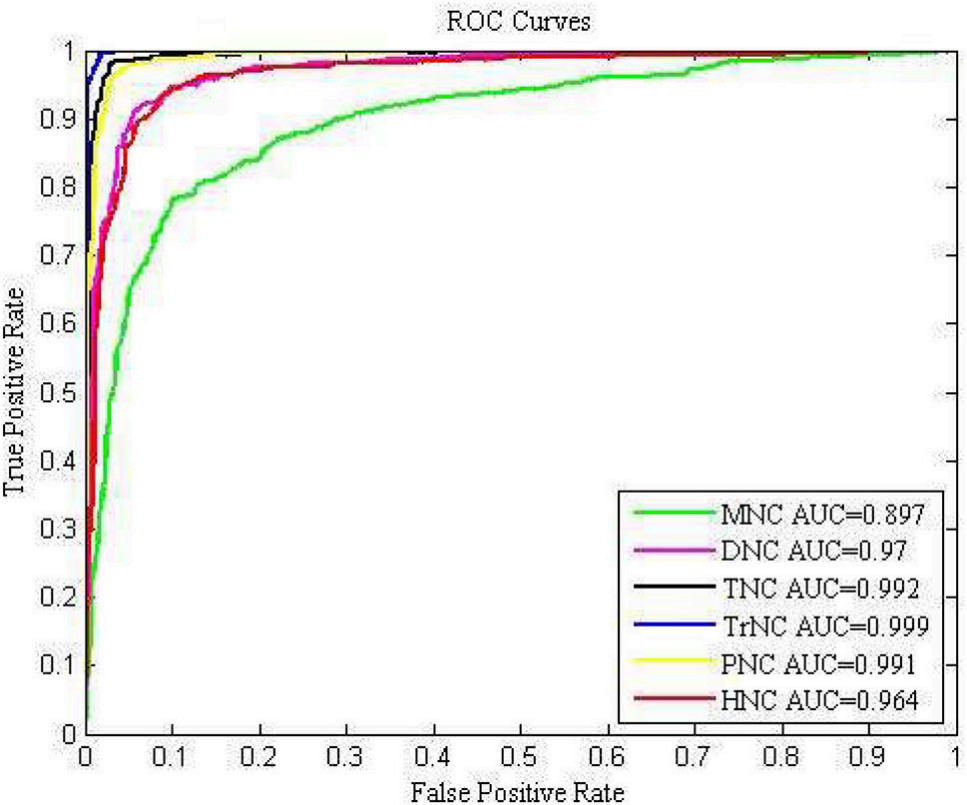
The ROC curves to assess the predictive performance based on different feature extraction methods.

To further investigate the overall prediction performance of each model, we showed the ROC curves and AUC values of different models for the 10-fold cross-validation in Figure 3. With the increase of *k*-mer value, the performance first increased and then decreased. Comparison demonstrated that the TrNC could produce the best results. Thus, the feature TrNC was adopted as the final model for Sc-ncDNAPred.

To further optimize the model, we performed multiple rounds of experiments on TrNC to select the appropriate subset of all 256 features (see Additional file 1: Table S1 for full details); however, the results showed no significant improvement in the corresponding performance. The possible reason is that the selected feature cannot burden enough information for the discrimination.

### Compositional analysis

**Figure 4:**
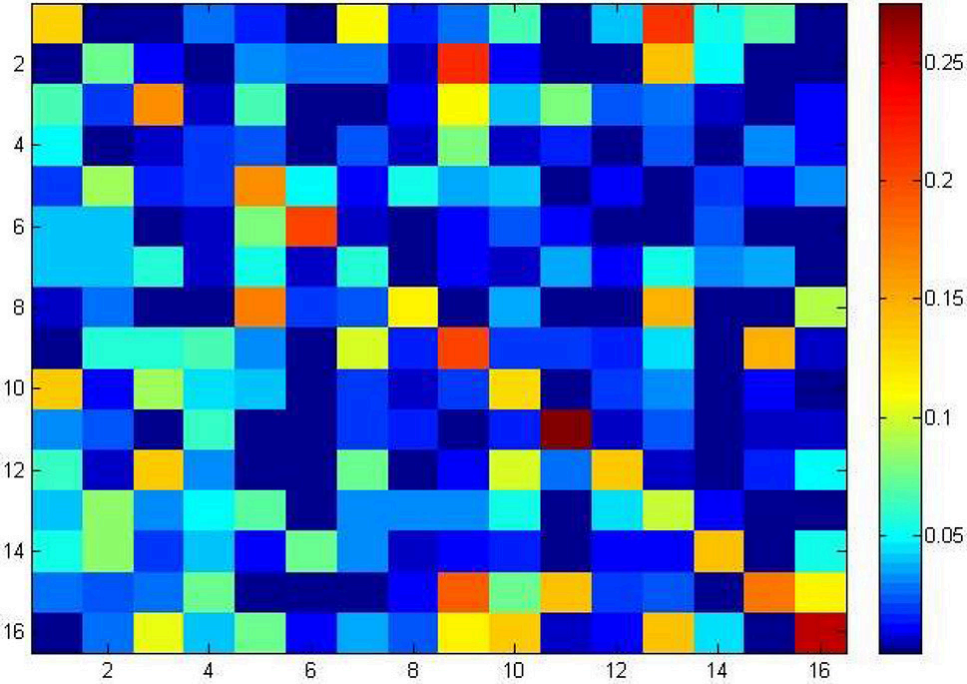
Heap map to illustrate the *F_score* values of 256 different tetramers to identify ncDNA and cDNA.

To understand the 256 different tetramers bias in ncDNAs and cDNAs, a heap map was provided in Figure 4. Each square in the heat map corresponds to the *F-score* value of one tetramer (see Table 2 for full details). Deep red in the heap map corresponds to a strong recognition ability.

**Table 2:** Rules of composition of heat map.

**AAAA**	**AAAC**	**AACA**	**AACC**	**ACAA**	**ACAC**	**ACCA**	**ACCC**	**CAAA**	**CAAC**	**CACA**	**CACC**	**CCAA**	**CCAC**	**CCCA**	**CCCC**
**AAAG**	**AAAT**	**AACG**	**AACT**	**ACAG**	**ACAT**	**ACCG**	**ACCT**	**CAAG**	**CAAT**	**CACG**	**CACT**	**CCAG**	**CCA**	**CCCG**	**CCCT**
**AAGA**	**AAGC**	**AATA**	**AATC**	**ACGA**	**ACGC**	**ACTA**	**ACTC**	**CAGA**	**CAGC**	**CATA**	**CATC**	**CCGA**	**CCGC**	**CCTA**	**CCTC**
**AAGG**	**AAGT**	**AATG**	**AATT**	**ACGG**	**ACGT**	**ACTG**	**ACTT**	**CAGG**	**CAG**	**CATG**	**CATT**	**CCGG**	**CCG**	**CCTG**	**CCTT**
**AGAA**	**AGAC**	**AGCA**	**AGCC**	**ATAA**	**ATAC**	**ATCA**	**ATCC**	**CGAA**	**CGAC**	**CGCA**	**CGCC**	**CTAA**	**CTAC**	**CTCA**	**CTCC**
**AGAG**	**AGAT**	**AGCG**	**AGCT**	**ATAG**	**ATAT**	**ATCG**	**ATCT**	**CGAG**	**CGAT**	**CGCG**	**CGCT**	**CTAG**	**CTAT**	**CTCG**	**CTCT**
**AGGA**	**AGGC**	**AGTA**	**AGTC**	**ATGA**	**ATGC**	**ATTA**	**ATTC**	**CGGA**	**CGGC**	**CGTA**	**CGTC**	**CTGA**	**CTGC**	**CTTA**	**CTTC**
**AGGG**	**AGGT**	**AGTG**	**AGTT**	**ATGG**	**ATGT**	**ATTG**	**ATTT**	**CGGG**	**CGGT**	**CGTG**	**CGTT**	**CTGG**	**CTGT**	**CTTG**	**CTTT**
**GAAA**	**GAAC**	**GACA**	**GACC**	**GCAA**	**GCAC**	**GCCA**	**GCCC**	**TAAA**	**TAAC**	**TACA**	**TACC**	**TCAA**	**TCAC**	**TCCA**	**TCCC**
**GAAG**	**GAAT**	**GACG**	**GACT**	**GCAG**	**GCAT**	**GCCG**	**GCCT**	**TAAG**	**TAAT**	**TACG**	**TACT**	**TCAG**	**TCAT**	**TCCG**	**TCCT**
**GAGA**	**GAGC**	**GATA**	**GATC**	**GCGA**	**GCGC**	**GCTA**	**GCTC**	**TAGA**	**TAGC**	**TATA**	**TATC**	**TCGA**	**TCGC**	**TCTA**	**TCTC**
**GAGG**	**GAGT**	**GATG**	**GATT**	**GCGG**	**GCGT**	**GCTG**	**GCTT**	**TAGG**	**TAGT**	**TATG**	**TATT**	**TCGG**	**TCGT**	**TCTG**	**TCTT**
**GGAA**	**GGAC**	**GGCA**	**GGCC**	**GTAA**	**GTAC**	**GTCA**	**GTCC**	**TGAA**	**TGAC**	**TGCA**	**TGCC**	**TTAA**	**TTAC**	**TTCA**	**TTCC**
**GGAG**	**GGAT**	**GGCG**	**GGCT**	**GTAG**	**GTAT**	**GTCG**	**GTCT**	**TGAG**	**TGAT**	**TGCG**	**TGCT**	**TTAG**	**TTAT**	**TTCG**	**TTCT**
**GGGA**	**GGGC**	**GGTA**	**GGTC**	**GTGA**	**GTGC**	**GTTA**	**GTTC**	**TGGA**	**TGGC**	**TGTA**	**TGTC**	**TTGA**	**TTGC**	**TTTA**	**TTTC**
**GGGG**	**GGGT**	**GGTG**	**GGTT**	**GTGG**	**GTGT**	**GTTG**	**GTTT**	**TGGG**	**TGGT**	**TGTG**	**TGTT**	**TTGG**	**TTGT**	**TTTG**	**TTTT**

**Figure 5:**
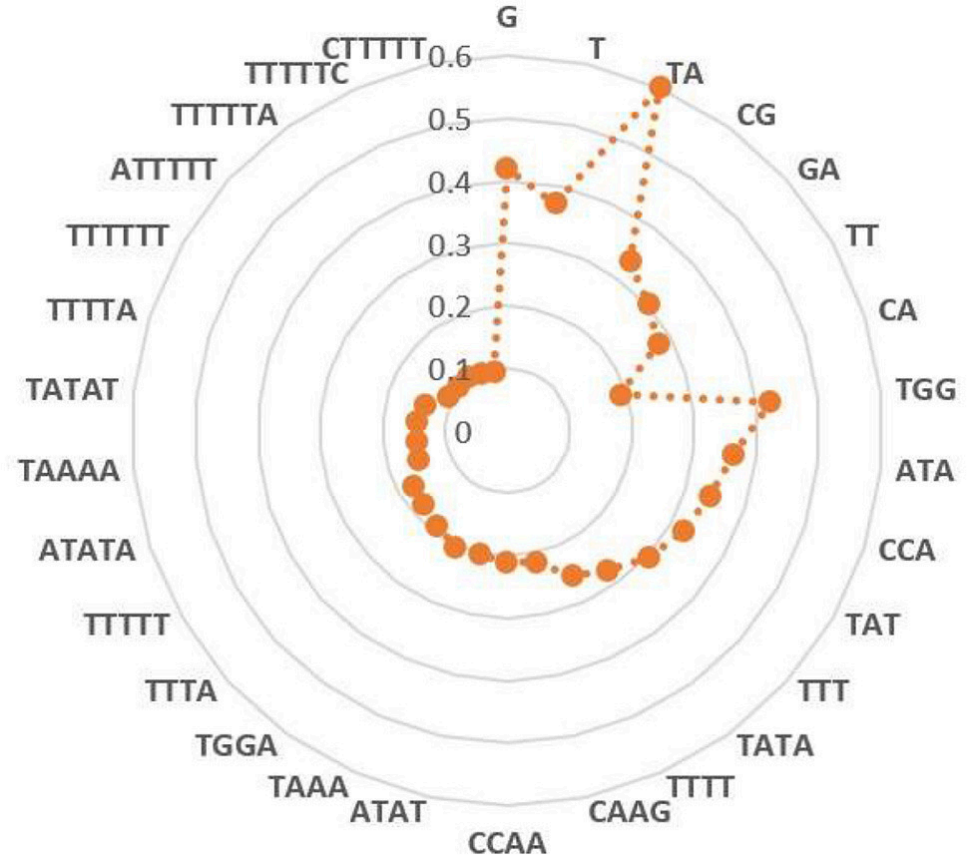
Key features of each *k*-mer composition selected by *F-score* method. Red color denotes *F-score* value of each feature.

Heap map analysis revealed that tetramers include TATA, TTTT, CAAG, CCAA, ATAT, TAAA, TGGA, TTTA, ATGG, ATAA, AATA, and CTGG are with the *F-score* values ranking top twelve in all tetramers. In addition, we also analyzed the other *k*-mer components based on the *F-score* method, respectively. Among them, the two key nucleotides G and T from MNC, the top five key dimer nucleotide composition (TA, CG, GA, TT, and CA) from DNC, (TGG, ATA, CCA, TAT, and TTT) from TNC, (TTTTT, ATATA, TAAAA, TATAT, and TTTTA) from PNC, and (TTTTTT, ATTTTT, TTTTTA, TTTTTC and CTTTTT) from HNC. These key features are presented in a radar diagram (Figure 5). The study of these key features can deepen the understanding of the overall structure of the genome, which not only promotes the annotation of the genome, but also promotes the study of biological evolution.

### Comparison with other classifiers

To the best of our knowledge, this is the first time that machine learning method has been used to identify ncDNA in S. cerevisiae. In order to further testify the superiority of proposed model Sc-ncDNAPred, the predictive results of it were compared with that of other powerful and widely used classifiers, i.e., k-Nearest Neighbor (KNN), Naïve Bayes, Random Forest, and J48 Tree as implemented in WEKA (Frank et al., 2004). The 10-fold cross validation results of these four classifier for identifying ncDNA in the same benchmark dataset were shown in Additional file 1: Table S2. The results showed that the four metrics as defined in Eq. 6 of the proposed model Sc-ncDNAPred are all higher than those of k-Nearest Neighbor (KNN), Naïve Bayes, Random Forest, and J48 Tree.

### Web-server

**Figure 6:**
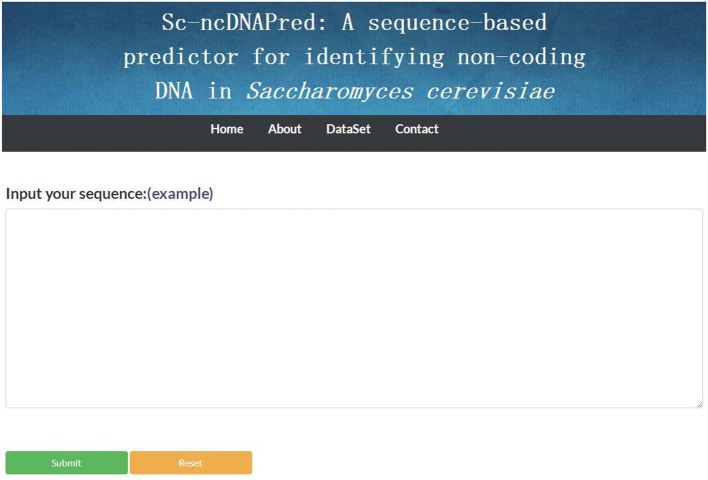
A semi-screenshot of the top page of the Sc-ncDNAPred web-server at: http://server.malab.cn/Sc_ncDNAPred/index.jsp.

Based on the benchmark dataset defined in Eq.1, a predictor called Sc-ncDNAPred was established, where “Sc” stands for *S. cerevisiae* and “Pred” stands for “Prediction.” For conveniences of users' community, a step-by-step guide about how to use the web-server is provided as follows:

Step 1. Open the web-server at: http://server.malab.cn/Sc_ncDNAPred/index.jsp, you will see the home page of Sc-ncDNAPred, as shown in Figure 6. Click the “About” button to see a brief introduction of the server.
Step 2. Paste the query DNA sequences into the input box. The input sequence should be in FASTA format. For the example of DNA sequences in FASTA format, click the “example” button top above the input box.
Step 3. Click on the “Submit” button to start the prediction. If the prediction result of a sequence is positive, its output is “ncDNA.” Otherwise, its output is “cDNA.”
Step 4. Click on the “DataSet” button to download the benchmark dataset.
Step 5. Click on the “Contact” button to contact us.

## Conclusions

DNA assembly technology needs a large number of target sequences of known information as data support. Non-coding DNA (ncDNA) sequences occupy most of the organism genomes, thus accurate recognizing of them is necessary. In this study, an efficient computational model was proposed to identify ncDNAs in *S. cerevisiae*. The tetramer nucleotide composition (TrNC) was adopted to extract features. The *F-score* method was used to analyze these feature vectors and find the key features. The high accuracy indicated that Sc-ncDNAPred was a powerful tool for predicting ncDNA. Finally, a free web-server was developed based on the proposed model. We hope that the predictor will provide convenience to most of scholars. Currently, annotations for the genomic sequences of most species are lacking or unavailable. To analyze the ncDNA data of these organisms, we can obtain data and methodological support in a cross-species manner from annotated species. For example, we could try to use the model built from *S. cerevisiae* dataset to analyze other species of bacteria that have not been explored in depth. In addition, we will also apply this computational model for the prediction of potential disease related non-coding DNA. In the future, we will apply this computational model for the prediction of potential disease related non-coding RNA (Chen and Huang, 2017; Chen et al., 2017a, 2018c,d; You et al., 2017).

## Author contributions

WH, QZ, and XL wrote the paper. XZ and YJ participated in preparation of the manuscript. QZ, WH, XL, XZ, and YJ participated in the research design. WH and QZ developed the web server. WH, YJ, XZ, XL, and QZ read and approved the final manuscript.

### Conflict of interest statement

The authors declare that the research was conducted in the absence of any commercial or financial relationships that could be construed as a potential conflict of interest.

## References

[B1] Baum E. B. (1995). Building an associative memory vastly larger than the brain. Science 268, 583–585. 7725109 10.1126/science.7725109

[B2] Byun H. Lee S. W. (2002). Applications of support vector machines for pattern recognition: a survey. In: Pattern Recognition With Support Vector Machines. Springer p (Niagara Falls, ON), 213–236.

[B3] Cao R. Wang Z. Wang Y. H. Cheng J. (2014). SMOQ: a tool for predicting the absolute residue-specific quality of a single protein model with support vector machines. BMC Bioinform. 15:120. 10.1186/1471-2105-15-120 24776231 PMC4013430

[B4] Carr P. A. Church G. M. (2009). Genome engineering. Nat. Biotechnol. 27, 1151–1162. 10.1038/nbt.1590 20010598

[B5] Chang C. C. Lin C. J. (2011). LIBSVM: a library for support vector machines. ACM Trans. Intell. Syst. Technol. 2:27 10.1145/1961189.1961199

[B6] Chen W. Ding H. Feng P. Lin H. Chou K. C. (2016). iACP: a sequence-based tool for identifying anticancer peptides. Oncotarget 7, 16895–16909. 10.18632/oncotarget.7815 26942877 PMC4941358

[B7] Chen X. Huang L. (2017). LRSSLMDA: laplacian regularized sparse subspace learning for MiRNA-disease association prediction. PLoS Comput. Biol. 13:e1005912. 10.1371/journal.pcbi.1005912 29253885 PMC5749861

[B8] Chen X. Huang L. Xie D. Zhao Q. (2018a). EGBMMDA: extreme gradient boosting machine for MiRNA-disease association prediction. Cell Death Dis. 9:3. 10.1038/s41419-017-0003-x 29305594 PMC5849212

[B9] Chen X. Huang Y. A. You Z. H. Yan G. Y. Wang X. S. (2018b). A novel approach based on KATZ measure to predict associations of human microbiota with non-infectious diseases. Bioinformatics 34:1440. 10.1093/bioinformatics/btx773 29253075

[B10] Chen X. Wang L. Qu J. Guan N. N. Li J. Q. (2018c). Predicting miRNA-disease association based on inductive matrix completion. Bioinformatics. 10.1093/bioinformatics/bty503 [Epub ahead of print]. 29939227

[B11] Chen X. Xie D. Wang L. Zhao Q. You Z. H. Liu H. (2018d). BNPMDA: bipartite network projection for MiRNA-disease association prediction. Bioinformatics. 10.1093/bioinformatics/bty333 [Epub ahead of print]. 29701758

[B12] Chen X. Xie D. Zhao Q. You Z. H. (2017a). MicroRNAs and complex diseases: from experimental results to computational models. Brief. Bioinform. 10.1093/bib/bbx130 [Epub ahead of print]. 29045685

[B13] Chen X. Yan C. C. Zhang X. You Z. H. (2017b). Long non-coding RNAs and complex diseases: from experimental results to computational models. Brief. Bioinform. 18, 558–576. 10.1093/bib/bbw060 27345524 PMC5862301

[B14] Chen X. Yan C. C. Zhang X. Zhang X. Dai F. Yin J.. (2016). Drug-target interaction prediction: databases, web servers and computational models. Brief. Bioinform. 17, 696–712. 10.1093/bib/bbv066 26283676

[B15] Chen X. X. Tang H. Li W. C. Wu H. Chen W. Ding H. (2016). Identification of bacterial cell wall lyases via pseudo amino acid composition. Biomed. Res. Int. 2016:1654623 10.1155/2016/1654623 27437396 PMC4942628

[B16] Chou K. C. (2001). Prediction of protein cellular attributes using pseudo-amino acid composition. Proteins Struct. Funct. Bioinform. 43, 246–55. 10.1002/prot.1035 11288174

[B17] Chou K. C. Shen H. B. (2008). Cell-PLoc: a package of Web servers for predicting subcellular localization of proteins in various organisms. Nat. Protoc. 3, 153–162. 10.1038/nprot.2007.494 18274516

[B18] Chou K. C. Zhang C. T. (1995). Prediction of protein structural classes. Crit. Rev. Biochem. Mol. Biol. 30, 275–349. 7587280 10.3109/10409239509083488

[B19] Davis J. (1996). Microvenus. Art J. 55, 70–74.

[B20] Eddy S. R. (2012). The C-value paradox, junk DNA and ENCODE. Curr. Biol. 22, R898–R899. 10.1016/j.cub.2012.10.002 23137679

[B21] Engler C. Gruetzner R. Kandzia R. Marillonnet S. (2009). Golden gate shuffling: a one-pot DNA shuffling method based on type IIs restriction enzymes. PloS ONE 4:e5553. 10.1371/journal.pone.0005553 19436741 PMC2677662

[B22] Erlich Y. Zielinski D. (2017). DNA Fountain enables a robust and efficient storage architecture. Science 355, 950–954. 10.1126/science.aaj2038 28254941

[B23] Frank E. Hall M. Trigg L. Holmes G. Witten I. H. (2004). Data mining in bioinformatics using Weka. Bioinformatics 20, 2479–2481. 10.1093/bioinformatics/bth261 15073010

[B24] Gibson D. G. Young L. Chuang R. Y. Venter J. C. Hutchison C. A. III. Smith H. O. (2009). Enzymatic assembly of DNA molecules up to several hundred kilobases. Nat Methods 6, 343–345. 10.1038/nmeth.1318 19363495

[B25] He W. Jia C. (2017). EnhancerPred2. 0: predicting enhancers and their strength based on position-specific trinucleotide propensity and electron–ion interaction potential feature selection. Mol BioSyst. 13, 767–74. 10.1039/c7mb00054e 28239713

[B26] He W. Jia C. Duan Y. Zou Q. (2018). 70ProPred: a predictor for discovering sigma70 promoters based on combining multiple features. BMC Syst. Biol. 12:44. 10.1186/s12918-018-0570-1 29745856 PMC5998878

[B27] Hearst M. A. Dumais S. T. Osuna E. Platt J. Scholkopf B. (1998). Support vector machines. IEEE Intell. Syst. Appl. 13, 18–28.

[B28] Horn S. Figl A. Rachakonda P. S. Fischer C. Sucker A. Gast A.. (2013). TERT promoter mutations in familial and sporadic melanoma. Science 339, 959–961. 10.1126/science.1230062 23348503

[B29] Hu H. Zhang L. Ai H. Zhang H. Fan Y. Zhao Q.. (2018). HLPI-ensemble: prediction of human lncRNA-protein interactions based on ensemble strategy. RNA Biol. 10.1080/15476286.15472018.11457935 [Epub ahead of print]. 29583068 PMC6152435

[B30] Hu H. Zhu C. Ai H. Zhang L. Zhao J. Zhao Q.. (2017). LPI-ETSLP: lncRNA-protein interaction prediction using eigenvalue transformation-based semi-supervised link prediction. Mol. Biosyst. 13, 1781–1787. 10.1039/c7mb00290d 28702594

[B31] Huang F. W. Hodis E. Xu M. J. Kryukov G. V. Chin L. Garraway L. A. (2013). Highly recurrent TERT promoter mutations in human melanoma. Science 339, 957–959. 10.1126/science.1229259 23348506 PMC4423787

[B32] Hubbard T. Barker D. Birney E. Cameron G. Chen Y. Clark L.. (2002). The ensembl genome database project. Nucleic Acids Res. 30, 38–41. 10.1093/nar/30.1.38 11752248 PMC99161

[B33] Jain M. Koren S. Miga K. H. Quick J. Rand A. C. Sasani T. A.. (2018). Nanopore sequencing and assembly of a human genome with ultra-long reads. Nat. Biotechnol. 36, 338–345. 10.1038/nbt.4060 29431738 PMC5889714

[B34] Jia C. He W. (2016). EnhancerPred: a predictor for discovering enhancers based on the combination and selection of multiple features. Sci. Rep. 6:38741. 10.1038/srep38741 27941893 PMC5150536

[B35] Jia C. Z. He W. Y. Yao Y. H. (2017). OH-PRED: prediction of protein hydroxylation sites by incorporating adapted normal distribution bi-profile Bayes feature extraction and physicochemical properties of amino acids. J. Biomol. Struct. Dyn. 35, 829–835. 10.1080/07391102.2016.1163294 26957000

[B36] Jia C. Z. Liu T. Wang Z. P. (2013). O-GlcNAcPRED: a sensitive predictor to capture protein O-GlcNAcylation sites. Mol. Biosyst. 9, 2909–2913. 10.1039/C3MB70326F 24056994

[B37] Khurana E. Fu Y. Chakravarty D. Demichelis F. Rubin M. A. Gerstein M. (2016). Role of non-coding sequence variants in cancer. Nat. Rev. Genet. 17, 93–108. 10.1038/nrg.2015.17 26781813

[B38] Kim C. S. Winn M. D. Sachdeva V. Jordan K. E. (2017). K-mer clustering algorithm using a MapReduce framework: application to the parallelization of the Inchworm module of Trinity. BMC Bioinform. 18:467. 10.1186/s12859-017-1881-8 29100493 PMC5670514

[B39] Kohavi R. (1995). A study of cross-validation and bootstrap for accuracy estimation and model selection. in Ijcai 95 Proceedings of the 14th International Joint Conference on Artificial Intelligence. Montreal, QC 1137–1145.

[B40] Li D. Luo L. Zhang W. Liu F. Luo F. (2016). A genetic algorithm-based weighted ensemble method for predicting transposon-derived piRNAs. BMC Bioinform. 17:329. 10.1186/s12859-016-1206-3 27578422 PMC5006569

[B41] Li M. Z. Elledge S. J. (2012). SLIC: a method for sequence-and ligation-independent cloning. Methods Mol. Biol. 852, 51–59. 10.1007/978-1-61779-564-0_5 22328425

[B42] Li W. Godzik A. (2006). Cd-hit: a fast program for clustering and comparing large sets of protein or nucleotide sequences. Bioinformatics 22, 1658–1659. 10.1093/bioinformatics/btl158 16731699

[B43] Liao Z. J. Li D. P. Wang X. R. Li L. S. Zou Q. (2018). Cancer diagnosis through IsomiR expression with machine learning method. Curr. Bioinform. 13, 57–63. 10.2174/1574893611666160609081155

[B44] Liu B. (2018). BioSeq-analysis: a platform for DNA, RNA, and protein sequence analysis based on machine learning approaches. Brief. Bioinform. 10.1093/bib/bbx165 [Epub ahead of print]. 29272359

[B45] Liu B. Fang L. Liu F. Wang X. Chen J. Chou K. C. (2015a). Identification of real microRNA precursors with a pseudo structure status composition approach. PLoS ONE 10:e0121501. 10.1371/journal.pone.0121501 25821974 PMC4378912

[B46] Liu B. Fang Y. Huang D. S. Chou K. C. (2018). iPromoter-2L: a two-layer predictor for identifying promoters and their types by multi-window-based PseKNC. Bioinformaitcs 34, 33–40. 10.1093/bioinformatics/btx579 28968797

[B47] Liu B. Liu F. Fang L. Wang X. Chou K. C. (2015b). repDNA: a Python package to generate various modes of feature vectors for DNA sequences by incorporating user-defined physicochemical properties and sequence-order effects. Bioinformatics 31, 1307–1309. 10.1093/bioinformatics/btu820 25504848

[B48] Liu B. Liu F. Wang X. Chen J. Fang L. Chou K. C. (2015c). Pse-in-One: a web server for generating various modes of pseudo components of DNA, RNA, and protein sequences. Nucleic Acids Res. 43, W65–W71. 10.1093/nar/gkv458 25958395 PMC4489303

[B49] Liu B. Wang S. Long R. Chou K. C. (2017a). iRSpot-EL: identify recombination spots with an ensemble learning approach. Bioinformatics 33, 35–41. 10.1093/bioinformatics/btw539 27531102

[B50] Liu B. Wu H. Zhang D. Wang X. Chou K. C. (2017b). Pse-analysis: a python package for DNA, RNA and protein peptide sequence analysis based on pseudo components and kernel methods. Oncotarget 8, 13338–13343. 10.18632/oncotarget.14524 28076851 PMC5355101

[B51] Liu B. Zhang D. Xu R. Xu J. Wang X. Chen Q.. (2014). Combining evolutionary information extracted from frequency profiles with sequence-based kernels for protein remote homology detection. Bioinformatics 30, 472–479. 10.1093/bioinformatics/btt709 24318998 PMC7537947

[B52] Liu L. M. Xu Y. Chou K. C. (2017). iPGK-PseAAC: identify lysine phosphoglycerylation sites in proteins by incorporating four different tiers of amino acid pairwise coupling information into the general PseAAC. Med. Chem. 13, 552–559. 10.2174/1573406413666170515120507 28521678

[B53] Liu X. Yu Y. Liu J. Elliott C. F. Qian C. Liu J. (2018). A novel data structure to support ultra-fast taxonomic classification of metagenomic sequences with k-mer signatures. Bioinformatics 34, 171–178. 10.1093/bioinformatics/btx432 29036588 PMC5870563

[B54] Luo L. Li D. Zhang W. Tu S. Zhu X. Tian G. (2016). Accurate prediction of transposon-derived piRNAs by integrating various sequential and physicochemical features. PloS ONE 11:e0153268. 10.1371/journal.pone.0153268 27074043 PMC4830532

[B55] Matias Rodrigues J. F. Schmidt T. S. B. Tackmann J. von Mering C. (2017). MAPseq: highly efficient k-mer search with confidence estimates, for rRNA sequence analysis. Bioinformatics 33, 3808–3810. 10.1093/bioinformatics/btx517 28961926 PMC5860325

[B56] Nasrabadi N. M. (2007). Pattern recognition and machine learning. J. Electr. Imaging 16:049901 10.18637/jss.v017.b05

[B57] Ni P. X. Dai W. K. Liu Y. F. Yang Z. Y. Zhou T. Liang S. Q. (2017). A novel method for better bacterialgenome assembly from illumina data. Curr. Bioinform. 12, 498–508. 10.2174/1574893610666150624171516

[B58] Orenstein Y. Pellow D. Marçais G. Shamir R. Kingsford C. (2017). Designing small universal k-mer hitting sets for improved analysis of high-throughput sequencing. PLoS Comput. Biol. 13:e1005777. 10.1371/journal.pcbi.1005777 28968408 PMC5645146

[B59] Pan Y. Liu D. Deng L. (2017a). Accurate prediction of functional effects for variants by combining gradient tree boosting with optimal neighborhood properties. PloS ONE 12:e0179314. 10.1371/journal.pone.0179314 28614374 PMC5470696

[B60] Pan Y. Wang Z. Zhan W. Deng L. (2017b). Computational identification of binding energy hot spots in protein-RNA complexes using an ensemble approach. Bioinformatics 34, 1473–1480. 10.1093/bioinformatics/btx822 29281004

[B61] Puente X. S. Beà S. Valdés-Mas R. Villamor N. Gutiérrez-Abril J. Martín-Subero J. I.. (2015). Non-coding recurrent mutations in chronic lymphocytic leukaemia. Nature 526, 519–524. 10.1038/nature14666 26200345

[B62] Qiao Y. Xiong Y. Gao H. Zhu X. Chen P. (2018). Protein-protein interface hot spots prediction based on a hybrid feature selection strategy. BMC Bioinform. 19:14. 10.1186/s12859-018-2009-5 29334889 PMC5769548

[B63] Qiu W. R. Sun B. Q. Xiao X. Xu Z. C. Chou K. C. (2016). iPTM-mLys: identifying multiple lysine PTM sites and their different types. Bioinformatics 32, 3116–3123. 10.1093/bioinformatics/btw380 27334473

[B64] Qiu W. R. Xiao X. Lin W. Z. Chou K. C. (2015). iUbiq-Lys: prediction of lysine ubiquitination sites in proteins by extracting sequence evolution information via a gray system model. J. Biomol. Struct. Dyn. 33, 1731–1742. 10.1080/07391102.2014.968875 25248923

[B65] Rangavittal S. Harris R. S. Cechova M. Tomaszkiewicz M. Chikhi R. Makova K. D.. (2018). RecoverY: k-mer-based read classification for Y-chromosome-specific sequencing and assembly. Bioinformatics 34, 1125–1131. 10.1093/bioinformatics/btx771 29194476 PMC6030959

[B66] Rheinbay E. Parasuraman P. Grimsby J. Tiao G. Engreitz J. M. Kim J.. (2017). Recurrent and functional regulatory mutations in breast cancer. Nature 547, 55–60. 10.1038/nature22992 28658208 PMC5563978

[B67] Senawi A. Wei H. L. Billings S. A. (2017). A new maximum relevance-minimum multicollinearity (MRmMC) method for feature selection and ranking. Pattern Recogn. 67, 47–61. 10.1016/j.patcog.2017.01.026

[B68] Shipman S. L. Nivala J. Macklis J. D. Church G. M. (2017). CRISPR–cas encoding of a digital movie into the genomes of a population of living bacteria. Nature 547, 345–349. 10.1038/nature23017 28700573 PMC5842791

[B69] Sleight S. C. Bartley B. A. Lieviant J. A. Sauro H. M. (2010). In-fusion biobrick assembly and re-engineering. Nucleic Acids Res. 38, 2624–2636. 10.1093/nar/gkq179 20385581 PMC2860134

[B70] Su Z. D. Huang Y. Zhang Z. Y. Zhao Y. W. Wang D. Chen W.. (2018). iLoc-lncRNA: predict the subcellular location of lncRNAs by incorporating octamer composition into general PseKNC. Bioinformatics. 10.1093/bioinformatics/bty508 [Epub ahead of print]. 29931187

[B71] Tang H. Chen W. Lin H. (2016). Identification of immunoglobulins using Chou's pseudo amino acid composition with feature selection technique. Mol. BioSyst. 12, 1269–1275. 10.1039/c5mb00883b 26883492

[B72] Tang H. Zhao Y. W. Zou P. Zhang C. M. Chen R. Huang P.. (2018). HBPred: a tool to identify growth hormone-binding proteins. Int. J. Biol. Sci. 14, 957–964. 10.7150/ijbs.24174 29989085 PMC6036759

[B73] Thomas C. A. Jr. (1971). The genetic organization of chromosomes. Annu. Rev. Genet. 5, 237–256. 16097657 10.1146/annurev.ge.05.120171.001321

[B74] Vinagre J. Almeida A. Pópulo H. Batista R. Lyra J. Pinto V.. (2013). Frequency of TERT promoter mutations in human cancers. Nat. Commun 4:2185. 10.1038/ncomms3185 23887589

[B75] Vogel F. (1964). A preliminary estimate of the number of human genes. Nature 201:847. 14161239 10.1038/201847a0

[B76] Warrens A. N. Jones M. D. Lechler R. I. (1997). Splicing by overlap extension by PCR using asymmetric amplification: an improved technique for the generation of hybrid proteins of immunological interest. Gene 186, 29–35. 9047341 10.1016/s0378-1119(96)00674-9

[B77] Wei H. L. Billings S. A. (2007). Feature subset selection and ranking for data dimensionality reduction. IEEE Trans. Pattern Anal. Mach. Intell. 29, 162–6. 10.1109/TPAMI.2007.11 17108391

[B78] Wei L. Liao M. Gao X. Zou Q. (2015). Enhanced protein fold prediction method through a novel feature extraction technique. IEEE Trans. Nanobiosci. 14, 649–659. 10.1109/TNB.2015.2450233 26335556

[B79] Wu Y. Li B. Z. Zhao M. Mitchell L. A. Xie Z. X. Lin Q. H.. (2017). Bug mapping and fitness testing of chemically synthesized chromosome X. Science 355:eaaf4706. 10.1126/science.aaf4706 28280152 PMC5679077

[B80] Xiao Y. Zhang J. Deng L. (2017). Prediction of lncRNA-protein interactions using HeteSim scores based on heterogeneous networks. Sci. Rep. 7:3664. 10.1038/s41598-017-03986-1 28623317 PMC5473862

[B81] Xie Z. X. Li B. Z. Mitchell L. A. Wu Y. Qi X. Jin Z.. (2017). “Perfect” designer chromosome V and behavior of a ring derivative. Science 355:eaaf4704. 10.1126/science.aaf4704 28280151

[B82] Xiong Y. Liu J. Wei D. Q. (2011). An accurate feature-based method for identifying DNA-binding residues on protein surfaces. Proteins 79, 509–517. 10.1002/prot.22898 21069866

[B83] Xu Q. Xiong Y. Dai H. Kumari K. M. Xu Q. Ou H. Y.. (2017). PDC-SGB: prediction of effective drug combinations using a stochastic gradient boosting algorithm. J. Theor. Biol. 417, 1–7. 10.1016/j.jtbi.2017.01.019 28099868

[B84] Yang H. Qiu W. R. Liu G. Q. Guo F. B. Chen W. Chou K. C.. (2018). iRSpot-Pse6NC: Identifying recombination spots in Saccharomyces cerevisiae by incorporating hexamer composition into general PseKNC. Int. J. Biol. Sci. 14, 883–891. 10.7150/ijbs.24616 29989083 PMC6036749

[B85] Yang H. Tang H. Chen X. X. Zhang C. J. Zhu P. P. Ding H.. (2016). Identification of secretory proteins in *Mycobacterium* tuberculosis using pseudo amino acid composition. Biomed. Res. Int. 2016:5413903. 10.1155/2016/5413903 27597968 PMC4997101

[B86] Yao Y. H. Li X. H. Geng L. L. Nan X. Y. Qi Z. H. Liao B. (2018). Recent progress in long noncoding RNAs prediction. Curr. Bioinformatics 13, 344–351. 10.2174/1574893612666170905153933

[B87] You Z. H. Huang Z. A. Zhu Z. Yan G. Y. Li Z. W. Wen Z. (2017). PBMDA: a novel and effective path-based computational model for miRNA-disease association prediction. PLoS Comput. Biol. 13:e1005455. 10.1371/journal.pcbi.1005455 28339468 PMC5384769

[B88] Zhang N. Yu S. Guo Y. Wang L. Wang P. Feng Y. (2018). Discriminating Ramos and Jurkat Cells with image textures from diffraction imaging flow cytometry based on a support vector machine. Curr. Bioinform. 13, 50–6., 10.2174/1574893611666160608102537

[B89] Zhang W. Bojorquez-Gomez A. Velez D. O. Xu G. Sanchez K. S. Shen J. P.. (2018). A global transcriptional network connecting noncoding mutations to changes in tumor gene expression. Nat. Genet. 50, 613–620. 10.1038/s41588-018-0091-2 29610481 PMC5893414

[B90] Zhang W. Liu J. Zhao M. Li Q. (2012a). Predicting linear B-cell epitopes by using sequence-derived structural and physicochemical features. Int. J. Data Min. Bioinform. 6, 557–569. 10.1504/IJDMB.2012.049298 23155782

[B91] Zhang W. Niu Y. Xiong Y. Zhao M. Yu R. Liu J. (2012b). Computational prediction of conformational B-cell epitopes from antigen primary structures by ensemble learning. PloS ONE 7:e43575. 10.1371/journal.pone.0043575 22927994 PMC3424238

[B92] Zhang W. Niu Y. Zou H. Luo L. Liu Q. Wu W. (2015). Accurate prediction of immunogenic T-cell epitopes from epitope sequences using the genetic algorithm-based ensemble learning. PloS ONE 10:e0128194. 10.1371/journal.pone.0128194 26020952 PMC4447411

[B93] Zhang W. Zhao G. Luo Z. Lin Y. Wang L. Guo Y.. (2017b). Engineering the ribosomal DNA in a megabase synthetic chromosome. Science 355:eaaf3981. 10.1126/science.aaf3981 28280149

[B94] Zhang W. Zhu X. Fu Y. Tsuji J. Weng Z. (2017a). Predicting human splicing branchpoints by combining sequence-derived features and multi-label learning methods. BMC Bioinform. 18(Suppl. 13):464. 10.1186/s12859-017-1875-6 29219070 PMC5773893

[B95] Zhou L. Q. Li R. Hu L. (2016). Enhanced prediction of small non-coding RNA in bacterial genomes based on improved inter-nucleotide distances of genomes. Curr. Bioinform. 11, 169–72. 10.2174/1574893611666160223201114

[B96] Zou Q. Liu W. Merler M. Ji R. (2016). Advanced learning for large-scale heterogeneous computing. Neurocomputing 217, 1–2. 10.1016/j.neucom.2016.06.009

