# Supplementary material for: Sc-ncDNAPred: A Sequence-Based Predictor for Identifying Non-coding DNA in Saccharomyces cerevisiae
Source: Front Microbiol. 2018 Sep 12;9:2174. doi: 10.3389/fmicb.2018.02174 (PMC6144933; doi:10.3389/fmicb.2018.02174)
Supplement: Supplementary file 1 [file Data_Sheet_1.ZIP › Additional file/Additional file 1.docx]

**The feature selection strategy of multiple rounds of experiments on TrNC**

First, the F-score values were calculated to rank the 256 features derived from TrNC, and then the F-score values were ranked from largest to smallest. To reduce the computational cost, we optimized the number of TrNC features from 1 to 256 using intervals of =10. SVM was employed to investigate the prediction performances of the selected features. The prediction performances are shown in **Table S1.** Finally, no redundant feature was removed in the TrNC features. Ultimately, 256 features from TrNC were kept to build the predictor Sc-ncDNAPred.

**Table S1** Predictive performance of TrNC features over different dimentions

| Dimension | Sn(%) | Sp(%) | ACC(%) | MCC |
| --- | --- | --- | --- | --- |
| 10 | 88.89 | 90.57 | 89.76 | 0.795 |
| 20 | 91.45 | 90.83 | 91.14 | 0.8228 |
| 30 | 92.23 | 91.65 | 91.93 | 0.8387 |
| 40 | 92.93 | 91.93 | 92.4 | 0.8482 |
| 50 | 93.04 | 92.28 | 92.66 | 0.8532 |
| 60 | 93.36 | 92.3 | 92.81 | 0.8563 |
| 70 | 93.53 | 9252 | 93.01 | 0.8602 |
| 80 | 93.73 | 92.76 | 93.23 | 0.8648 |
| 90 | 93.84 | 92.63 | 93.23 | 0.8646 |
| 100 | 94.15 | 92.54 | 93.34 | 0.8669 |
| 110 | 94.27 | 92.52 | 93.38 | 0.8677 |
| 120 | 94.37 | 92.7 | 93.53 | 0.8707 |
| 130 | 94.26 | 93.0 | 93.62 | 0.8724 |
| 140 | 94.31 | 93.32 | 93.81 | 0.8762 |
| 150 | 94.28 | 93.55 | 93.91 | 0.8782 |
| 160 | 94.61 | 93.78 | 94.18 | 0.8836 |
| 170 | 94.42 | 94.08 | 94.24 | 0.8849 |
| 180 | 94.82 | 94.6 | 94.71 | 0.8942 |
| 190 | 97.64 | 98.25 | 97.95 | 0.9589 |
| 200 | 97.78 | 98.2 | 98.0 | 0.9599 |
| 210 | 97.77 | 98.29 | 98.03 | 0.9606 |
| 220 | 97.85 | 98.4 | 98.14 | 0.9627 |
| 230 | 97.79 | 98.39 | 98.09 | 0.9619 |
| 240 | 97.82 | 98.5 | 98.17 | 0.9633 |
| 250 | 98.04 | 98.56 | 98.31 | 0.9661 |
| 256 | 98.09 | 98.54 | 98.32 | 0.9664 |

**Table S2** Comparison of different classifiers for identifying ncDNA by 10-fold cross-validation on the benchmark dataset

| Classifier | Sn(%) | Sp (%) | Acc(%) | MCC |
| --- | --- | --- | --- | --- |
| KNN(*k*=2) | 86.7 | 88.4 | 87.6 | 0.752 |
| Naïve Bayes | 75.6 | 85.0 | 80.4 | 0.609 |
| J48 | 85.8 | 86.1 | 86.0 | 0.719 |
| Random Forest | 96.0 | 88.5 | 92.2 | 0.846 |
